# Supplementary material for: Complexome profiling of the Chlamydomonas psb28 mutant reveals TEF5 as an early PSII assembly factor
Source: Plant Cell. 2025 Mar 17;37(6):koaf055. doi: 10.1093/plcell/koaf055 (PMC12164590; doi:10.1093/plcell/koaf055)
Supplement: koaf055_Supplementary_Data [file koaf055_supplementary_data.zip › psb28_interactive.zip!/plots/intensity/qualityPlots/peptides/CGL105_Cre12.g507400.t1.2.html]

### Cre12.g507400.t1.2

Trivial name: CGL105  
SeqCoverage [median]: 30.00  
Mass: 76.57 kDa  
  
 

| Sequence | MissedCleavage | Mass [Da] | wtIntensity | muIntensity | Score |
| --- | --- | --- | --- | --- | --- |
| AEDLTTGHVGAPNPAVEIK | 0 | 1917.98 | 9009.80 | 16642.86 | 126.84 |
| AQAQATAQQR | 0 | 1071.54 | 1144.89 | 1013.04 | 141.20 |
| FQEELDAMYAAMPATH | 0 | 1823.79 | 0.00 | 1444.39 | 108.68 |
| GAVLTHGNLISNAAGTATLLTESSPGDR | 0 | 2722.39 | 0.00 | 1000.05 | 44.26 |
| GPSVFAGYYKDEAQTR | 1 | 1787.85 | 1556.57 | 7292.38 | 101.36 |
| HLSYLPLAHIYER | 0 | 1610.86 | 0.00 | 1391.18 | 40.07 |
| IENTYTR | 0 | 895.44 | 453.11 | 1445.02 | 77.74 |
| LAQGEYIAPEK | 0 | 1217.63 | 3137.62 | 5069.01 | 134.13 |
| LAQGEYIAPEKIENTYTR | 1 | 2095.06 | 2000.80 | 8231.12 | 88.43 |
| LGGELK | 0 | 615.36 | 2265.30 | 0.00 | 85.31 |
| LMTLEAVEGLGR | 0 | 1287.69 | 728.15 | 2583.12 | 98.94 |
| LVDIPEMGYTAR | 0 | 1363.68 | 0.00 | 5129.45 | 87.30 |
| SATSPFQLVSTYPPPADHVR | 0 | 2169.09 | 0.00 | 14797.91 | 93.85 |
| TALGSGLMQLGLQPK | 0 | 1512.83 | 2343.62 | 12086.25 | 151.26 |
| TLHDNLEYAASK | 0 | 1360.66 | 2580.64 | 6890.97 | 114.86 |
| VMLAVQTGSPLAR | 0 | 1341.74 | 0.00 | 3649.63 | 123.75 |
|  |  |  | 25220.5 | 88666.4 |  |
